# Reduction of thyroid radioactive iodine exposure by oral administration of cyclic oligosaccharides

**DOI:** 10.1038/s41598-023-34254-0

**Published:** 2023-04-28

**Authors:** Kodai Nishi, Masahiro Hirota, Shogo Higaki, Shinya Shiraishi, Takashi Kudo, Naoki Matsuda, Shigeki Ito

**Affiliations:** 1 Department of Radioisotope Medicine, Atomic Bomb Disease Institute, Nagasaki University, Nagasaki, Japan; 2 Research Center for Supports to Advanced Sciences, Shinshu University, Matsumoto, Japan; 3 Isotope Science Center, The University of Tokyo, Tokyo, Japan; 4 Department of Radiology, Faculty of Life Sciences, Kumamoto University, Kumamoto, Japan; 5 Department of Radiation Biology and Protection, Atomic Bomb Disease Institute, Nagasaki University, Nagasaki, Japan; 6 Department of Medical Radiation Sciences, Faculty of Life Sciences, Kumamoto University, 4-24-1 Kuhonji Chu-ku, Kumamoto City, 862-0976 Japan

**Keywords:** Biochemistry, Biotechnology

## Abstract

Alpha-cyclodextrin, a six d-glucose cyclic oligosaccharide, has several applications in food and pharmaceuticals, but has also been reported to retain iodine in a stable manner for 16 months. Radioactive iodine, which may cause thyroid cancer and hypofunction, must be properly managed. If the absorption of radioactive iodine is suppressed, it can be expected to lead to a reduction in thyroid exposure. This study clarified the inhibition of radioactive iodine absorption by the oral administration of α-cyclodextrin in a murine model using direct measurement of single photon emission computed tomography. The uptake of radioactive iodine into the thyroid gland in mice administered with radioactive iodine and an α-cyclodextrin solution was approximately 40% lower after 24 h. The finding that oral uptake of α-cyclodextrin has an inhibitory effect on the transfer of radioactive iodine to the thyroid gland has potential for application in many fields such as food, pharmaceuticals, nuclear emergency preparedness, and medicine.

## Introduction

Iodine is the main ingredient of thyroid hormones (triiodothyronine: T3 and thyroxine: T4)^1^. When iodine is orally ingested, it is mainly absorbed in the form of iodide from the small intestine and transferred into the blood^1–7^. In addition, when iodine is inhaled, it either enters the blood from the upper respiratory tract and lungs or is absorbed from the small intestine and enters the blood^2,6^. Because the thyroid gland produces thyroid hormones, it selectively absorbs iodine ions from the blood^1^. The iodine ions absorbed in the thyroid gland are oxidized and retained within the follicles as thyronine, a precursor of thyroid hormones^1^.

Cyclodextrins (CD) are cyclic oligosaccharides composed of six to eight d-glucose molecules and are usually made from corn and potato starches^8^. CD composed of six, seven, and eight d-glucose molecules are distinguished as α-, β-, and γ-CD, respectively^8^. The internal diameters of α-, β-, and γ-are approximately 0.6, 0.8, and 1.0 nm^9^. The outer aspect of the cyclic structure of CD is hydrophilic due to the presence of hydroxyl groups of glucose, and the inner aspect is hydrophobic due to the presence of the glucose methine group^10^. Therefore, CD comprise hydrophobic substances with a size corresponding to the inner diameter, such as iodine or iodide anions, into the ring structure by intermolecular force, forming an inclusion complex^9–14^. The α-CD has been reported to retain iodine in a stable manner for 16 months^15^. In comparing α-CD and β-CD, it has also been reported that α-CD retains more iodine^16^. It has also been reported that α-CD retains radioactive iodine in low iodine concentration solutions (below 10^−8^ mol cm^−3^), which is found in radioactive sources and radioactive wastes, and has the effect of suppressing volatilization; the retention of radioactive iodine does not change even if α-CD is exposed to high-dose radiation^17^.

Orally ingested α-CD has been reported to slightly increase the gastric residence time of food and drink from 135 to 195 min after ingestion^18^. Furthermore, very little absorption occurs in the gastrointestinal tract as it is hardly degraded until it reaches the large intestine^19^. This property allows α-CD to be used to prevent volatilization of substances, to promote sustained release of pharmaceutical ingredients, solubilize poorly water-soluble substances, remove unwanted components, and is often used as a food additive for flavoring/smell correction and emulsification^19–24^. In addition, α-CD is also used as a functional food, such as in water-soluble dietary fiber, and in the suppression of sugar and fat absorption^18,25–31^. The α-CD is a highly safe substance for internal administration, with the upper limit of acceptable daily intake not clearly defined in the safety evaluation of food additives by the Joint FAO/WHO Expert Committee on Food Additives^32^.

It was hypothesized that iodine absorption from the small intestine could be suppressed based on the following facts: (1) iodine is mainly absorbed from the small intestine^2,6^, (2) α-CD forms an inclusion complex with iodine^9,10^, and (3) α-CD is hardly degraded until it reaches the large intestine^19^. When iodine absorption is suppressed, it is a concern that it will affect the thyroid uptake rate test and internal radioactive iodine therapy performed in the field of nuclear medicine. The thyroid uptake rate test is a test in which capsules of radioactive iodine are orally administered; whether the thyroid gland is functioning normally or not is evaluated by measuring the amount of transfer to the thyroid gland^33^. Internal radioactive iodine therapy is performed for the treatment of Graves' disease and thyroid cancer. It is a therapeutic method of radioactive iodine capsule administration that destroys excess thyroid and cancer tissue by internal radiation released from radioactive iodine that has accumulated in the thyroid gland and thyroid cancer metastatic sites. The ^123^I is mainly used in thyroid uptake rate tests, and ^131^I is used in internal radioactive iodine therapy^34^. Radioactive iodine administered for therapeutic or diagnostic purposes must be properly managed, given that the onset of thyroid cancer and hypothyroidism increases with inadvertent radiation exposure^35,36^.

The following three cases are conceivable as causes of the inadvertent ingestion of radioactive iodine. The first is the case in which facility workers and surrounding residents intake ^131^I released into the environment due to a nuclear disaster^35–37^. The second is inadvertent contact of medical staff and the patient's family with a patient who has been administered radioactive iodine^38–40^. The third is the case in which radiation workers ingest radioactive iodine due to the mishandling of radiation sources and contaminants used in research, medicine, and industry. If the absorption of radioactive iodine is suppressed in these cases, it can be expected to lead to a reduction in thyroid exposure. The inhibitory effect of α-CD on radioactive iodine may contribute to radiation safety by reducing thyroid exposure through the ingestion of food or drink, which is safer than pharmaceuticals. This means that α-CD, which is also used as a food additive and is recognized as highly safe, may contribute to the reduction of thyroid exposure by radioactive iodine.

The purpose of this study was to elucidate the inhibitory effect of α-CD on radioactive iodine absorption from the gastrointestinal tract by comparing the pharmacokinetic differences between radioactive iodine contained in α-CD and radioactive iodine not contained in α-CD in mice.

## Methods

### Drug adjustment

Two types of radioactive iodine, ^123^I and ^131^I, were used in the experiment. Na^123^I and Na^131^I were purchased from PDR Pharma Co. (Tokyo, Japan). An amount of 50 MBq of ^123^I was dissolved in 100 µL of saline and mixed with a 150 µL, 5% weight concentration of α-CD solution. An amount of 15 MBq of ^131^I was dissolved in 100 µL of saline and mixed with a 150 µL, 5% weight concentration of α-CD solution.

### Thyroxine value measurement

Animal studies were performed in accordance with the recommendations of the Fundamental Guidelines for Proper Conduct of Animal Experiments and Related Activities in Academic Research Institutions under the jurisdiction of the Ministry of Education, Culture, Sport, Science and Technology, Japan. The Animal Care and Use Committee of Nagasaki University approved all experimental protocols (approval number: 2204271787). All experiments with mice were performed in accordance with the ARRIVE guidelines.

All animal experiences were used the same lot male mice (ddY, 5 weeks old; body weight (BW), 22–26 g) purchased from Japan SLC, Inc. (Shizuoka, Japan).

Blood samples of ten ddY-mice were collected from the posterior vena cava under isoflurane inhalational anesthesia. The blood was centrifuged at 10,000 rpm at 4 °C for 3 min and the serum obtained was frozen at below 75 °C. Thyroid hormone (thyroxine: T4) concentration was measured by enzyme-linked immunosorbent assay (ELISA) method^41,42^ using a Mouse/Rat Thyroxine ELISA kit (Calbiotech, El Cajon, CA, USA). The T4 values of the mice used in the current study were within the expected range (between 4 and 12 µg/dL; euthyroid; Table 1)^43,44^.

**Table 1:** Body weight of mice and thyroxine (T4) value before the start of the experiment.

No	Body weight (g)	T4 (μg/dL)
1	25.1	5.2
2	23.9	5.5
3	24.3	5.9
4	25.0	5.2
5	25.9	6.4
6	25.4	4.8
7	23.8	5.8
8	22.0	6.2
9	23.6	5.5
10	24.8	4.8
Means	24.4 ± 1.1	5.5 ± 0.5

### Single-photon emission computed tomography (SPECT) imaging

One week before the start of the imaging experiment, six animals were reared by switching to a low-iodine diet (LID) (CLEA Diet No.11 CE-2, CLEA Japan, Inc., Tokyo, Japan). All imaging studies were performed using the Triumph combined positron emission tomography (PET)/single photon emission computed tomography (SPECT)/computed tomography (CT) system (TriFoil Imaging, Chatsworth, CA, USA).

An amount of 250 µL of radioactive α-CD solution was intragastrically administered to each mouse, and SPECT imaging was carried out under inhalational anesthesia (1.5% isoflurane) 3, 6, and 24 h after administration. A control group was administered a solution in which the α-CD solution was replaced with saline, and imaging was performed in the same manner. SPECT acquisitions were performed for 21 min with 64 views over 360°, 20 s/projection, using a 60-mm radius of rotation. After SPECT, CT was performed for anatomical reference. SPECT data were reconstructed using a three dimensional (3D)-maximum-likelihood expectation maximization algorithm (50 iterations). The CT and SPECT data were processed and analyzed using OsiriX MD (Pixmeo, Geneva, Switzerland).

### SPECT image analysis

**Figure 1:**
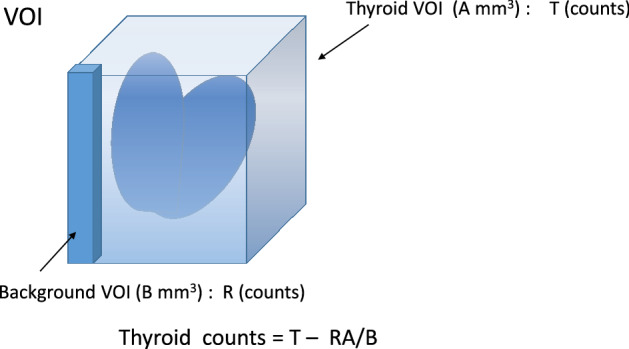
The thyroid gland and background regions were obtained in each slice of the single-photon emission computed tomography (SPECT) image, and by summation, the count value was obtained as a three-dimensional volume of interest (VOI). Thyroid counts = T − RA/B; Thyroid VOI (A mm^3^), T (counts), background VOI (B mm^3^), R (counts).

The thyroid gland and background regions were obtained in each slice of the SPECT image, and by summation, the count value was obtained as a three-dimensional volume of interest (VOI) (Fig. 1). The thyroid uptake was calculated from the obtained counts and administered dose using Eq. (1):

1 $${\text{Thyroid uptake}}\;\left( \% \right) = \left( {\text{thyroid counts}} \right) \times {\text{CF}} \times 100\;\left( {{\text{MBq}}} \right)/{\text{administered dose}}\;\left( {{\text{MBq}}} \right),$$

where CF (counts/MBq) is the count activity conversion factor. The CF value was determined by using standard dose SPECT counts. CF was calculated from the counts and radioactivity of the standard radiation source by placing a radiation source with a known fluid volume and radioactivity for each mouse SPECT on the back, which did not affect the thyroid SPECT counts of the mice. The inhibitory effect of α-CD on absorption of radioactive iodine from the gastrointestinal tract was clarified by comparing the chronological thyroid uptake values of the control and α-CD administration groups.

### Statistical analysis

All data are expressed as mean ± standard deviation (SD) and were statistically analyzed by t-test using MedCalc Statistical Software version 20.115 (MedCalc. Software Ltd, Ostend, Belgium; https://www.medcalc.org; 2020).

## Results

**Figure 2:**
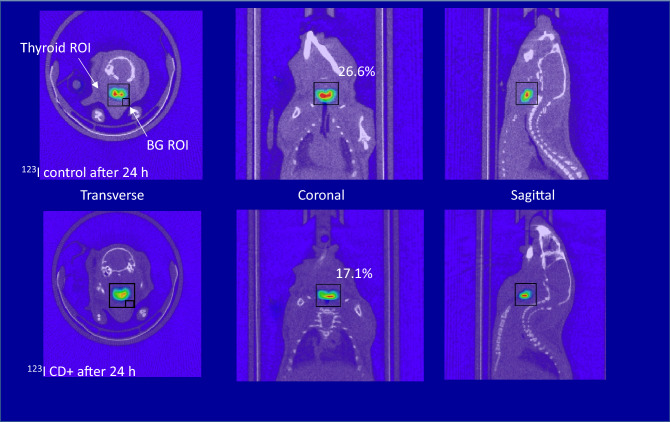
The 24-h thyroid single-photon emission computed tomography (SPECT) images of Na^123^I and α-CD administered mice, and control mice with normal thyroid function (with α-CD solution replaced with saline).

**Figure 3:**
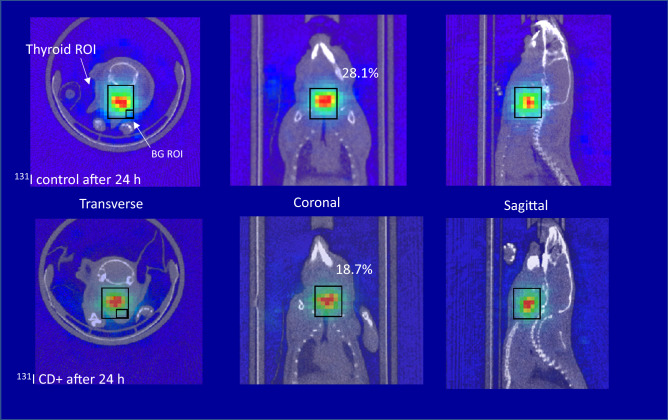
The 24-h thyroid single-photon emission computed tomography (SPECT) images of Na^131^I + α-CD-administered mice and control mice with normal thyroid function (in which the α-CD solution was replaced with saline).

Figure 2 shows the 24-h thyroid SPECT images of Na^123^I + α-CD administered mice and control mice with normal thyroid function in which the α-CD solution was replaced with saline. The accumulation of Na^123^I in the thyroid gland of α-CD-administered mice was also visually confirmed to be lower than that of the control. Figure 3 shows the 24-h thyroid SPECT images of Na^131^I + α-CD-administered mice and the control mice (with normal thyroid function, in which the α-CD solution was replaced with saline). The resolution of the ^131^I thyroid images was inferior to the ^123^I thyroid images.

**Figure 4:**
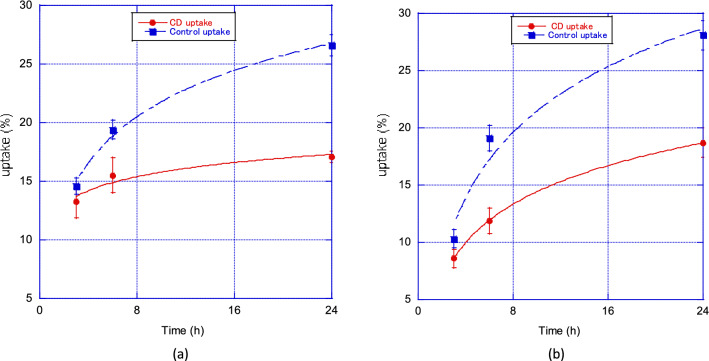
Changes in thyroid uptake values in the (**a**) Na^123^I + α-CD-administration and control groups, and (**b**) Na^131^I + α-CD-administration and control groups.

**Figure 5:**
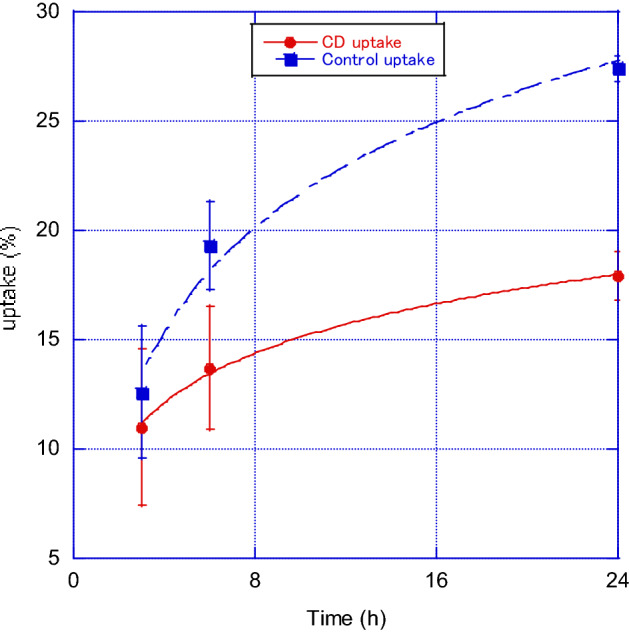
Temporal changes in radioactive iodine uptake rate with and without α-CD administration.

Figure 4a and b show the changes in thyroid uptake in the Na^123^I + α-CD-administration and control groups, and the Na^131^I + α-CD and control groups, respectively. In the Na^123^I group, the uptake values of the control after 3, 6, and 24 h were 14.6 ± 0.7%, 19.4 ± 0.8%, and 26.6 ± 0.9%, whereas in the α-CD administration group, they were 13.3 ± 2.4%, 15.5 ± 3.1%, and 17.1 ± 0.5% and the 24-h uptake was approximately 40% lower than that of the control. Na^131^I showed the same tendency as Na^123^I, and the uptake values of the control after 3, 6, and 24 h were 10.3 ± 0.8%, 19.1 ± 1.1%, and 28.1 ± 1.3%, whereas in the α-CD administration group, they were 8.6 ± 0.8%, 11.9 ± 1.1%, and 18.7 ± 1.3%; the 24-h values were approximately 40% lower than that of the control. No difference in uptake due to differences in Na^123^I and Na^131^I nuclides was observed. Figure 5 shows the temporal changes in radioactive iodine uptake with and without α-CD administration. Uptake values of the control group after 3, 6, and 24 h were 12.6 ± 3.0, 19.3 ± 0.2, and 27.4 ± 0.6%, while those in the α-CD administration group were 11.0 ± 3.6, 13.7 ± 2.8, and 17.9 ± 1.1% and the 24-h uptake was approximately 40% lower than that of the control.

## Discussion

Iodine-123 emits a 159 keV γ-ray, which is suitable for SPECT imaging and analysis. Iodine-131 emits a maximum of 606 keV β-rays and mainly emits 356 keV γ-rays, which increases scattered and penetrative radiation. For this reason, the resolution of the ^131^I SPECT image is inferior to the ^123^I SPECT image. Additionally, the accuracy of the ^131^I thyroid quantification can be inferior to that of ^123^I. In this study, the accuracy of the ^131^I thyroid value using the SPECT analysis was optimized through the procedure of the ^123^I thyroid quantification (VOI size, VOI location, background size, and background location settings) as shown in Figs. 1, 2 and 3. The uptake values of Na^123^I and Na^131^I nuclides demonstrated similar accuracy.

The α-CD and β-CD selectively include iodine and are hardly degraded in the gastrointestinal tract until the small intestine where iodine is absorbed. The γ-CD, on the other hand, is degraded in the stomach by amylase^24^. Hence, it is considered that radioactive iodine absorption in the gastrointestinal tract is inhibited by α-CD and β-CD. In the safety evaluation of food additives by the Joint FAO/WHO Expert Committee on Food Additives, the upper limit of acceptable daily intake of β-CD is recommended to be 5 mg/kg BW/day and is not clearly defined^37,45^. Therefore, the inhibitory effect of α-CD on gastrointestinal absorption of radioactive iodine was clarified by comparing the thyroid uptake rates of radioactive iodine + α-CD, and control (in which the α-CD solution was replaced by saline) in a murine model. In the α-CD administration group, the 24-h uptake rate was approximately 40% lower than that of the control group. Therefore, α-CD administration was shown to inhibit gastrointestinal absorption of radioactive iodine.

When radioactive iodine solution was ingested orally, iodine could exist in various chemical forms. However, these were absorbed as iodide ions (I^−^). Absorbed I^−^ binds to tyrosine, which becomes T3 and T4. Subsequently, iodine was released as I^−^ by deiodinase in the body. The released I^−^ is oxidized and produces I_2_, which can further combine with I^−^ to form I_3_^−^. α-CD is sufficient if the quantities of I^−^, I_2_, and I_3_^−^ can be included^11,13,46^. Therefore, α-CD inhibits iodine absorption in the gastrointestinal tract.

Alpha-cyclodextrin is already added to foods and pharmaceuticals for various purposes^18–31^. Oral administration of CD at high doses (1000 mg/kg BW/day) or more can cause reversible diarrhea and cecal enlargement in animals^47^. In the current study, the concentrations and doses of α-CD were determined in terms of mouse weight, based on the components contained in 500 mL of commercially-available, healthy drinking water, in order to maintain normal health^48^. The amount of α-CD used in the experiment was considered to have zero health effects on living organisms.

When the iodine concentration in the blood is high, the synthesis of thyroid hormones is temporarily suppressed, and the uptake of iodine from the blood into the thyroid gland is suppressed^33^. In this study, the uptake of iodine in the normal thyroid gland was standardized using the low-iodine diet. Therefore, it is highly possible that the iodine-inhibiting effect is less than experimental data from the current study in the normal consumption of iodine-containing foods.

In the present study, to highlight the differences in the kinetics of iodine included in CD, the method for the effective including rate between CD and iodine was used. The presence of CD in the gastrointestinal tract was found to be able to alter iodine kinetics. We performed a similar study in mice pre-treated with CD as the next step.

Animal experiments are generally conducted with the lots and sexes aligned but it is common practice not to compare and verify the results of male and female animals unless the phenomenon is clearly influenced by differences in sex. In this case, only males were evaluated as a matter of convention, but it can be assumed that the same results would be obtained for females as well because the absorption of radioactive iodine in the gastrointestinal tract with normal thyroid function is more likely to have similar results regardless of sex.

As the thyroid uptake ratio depends on the dietary components ingested, subjects were standardized as having normal thyroid function and a low iodine diet based on the radioiodine thyroid uptake measurement method used in medicine^49^. Therefore, the thyroid uptake ratio under a normal diet (containing iodine) is considered to be lower than the results of this experiment. As a next step, we are conducting a similar study in mice fed a regular iodine diet.

The optimal concentration and amount of α-CD as an absorption inhibitor when radioactive iodine is orally ingested, and the timing of administration, need to be examined in detail in the future. In addition, it is necessary to examine the inhibition of absorption after inhalation of radioactive iodine from the lungs in detail. The current experiment clarified the inhibitory effect of radioactive iodine absorption by the oral administration of α-CD. The α-CD is also known to have an inhibitory effect on the absorption of cholesterol and triglycerides as it includes lecithin, which aids lipid absorption from the small intestine^30,31^. Therefore, there is scope for further examination of the effect of internal substances such as lecithin, which may compete with iodine for α-CD inclusion, on the inhibition of radioactive iodine absorption.

Prior to the thyroid uptake tests and radioactive iodine therapy, which are performed in the field of nuclear medicine, it is necessary to understand the absorption of foods containing α-CD, in addition to pretreatments such as low-iodine diets, and to take measures such as restricting the uptake of these foods.

As a preventive measure against thyroid exposure in, for example, the event of a nuclear power plant accident, the transfer of radioactive iodine to the thyroid gland is prevented by filling the thyroid gland with non-radioactive iodine by taking a stable iodine agent in advance^43^. However, frequent administration in children, pregnant women, lactating women, and the elderly; the long-term administration of excessive amounts; and iodine administration to patients with hypocomplementemic vasculitis, dermatitis herpetiformis, and iodine allergy should be avoided^50^. In euthyroid adults receiving iodine-sufficient diets (250 mg d21 in the current analysis), at least 30 mg KI administered up to 48 h before ^131^I exposure can block over 95% thyroid uptake and reduce the thyroid absorbed dose^51^. For such subjects, administration of α-CD is expected to reduce thyroid exposure. In addition, stable iodine agents are not approved for constant or multiple administration, and those who deal with radioactive iodine on a daily basis, such as medical staff and radiation workers, cannot take them to prevent thyroid exposure^49^. The α-CD, which is also used as a food additive, has a great advantage in that it can be taken prophylactically. Thus, utilization of α-CD, which has the effect of inhibiting the transfer of radioactive iodine to the thyroid gland after oral uptake, can be implemented in a wide range of fields such as medicine and nuclear emergency preparedness. Applications in other fields are expected in the future.

## Conclusion

The uptake of radioactive iodine into the thyroid gland in mice administered with radioactive iodine + α-CD solution, and control mice (in which the α-CD solution was replaced with saline) decreased by approximately 40% in the α-CD group than the non-α-CD group after 24 h. The finding that oral uptake of α-CD has an inhibitory effect on the transfer of radioactive iodine to the thyroid gland has potential for application in many fields such as food, pharmaceuticals, nuclear emergency preparedness, and medicine.

## Data Availability

The datasets generated and/or analyzed during the current study are available from the corresponding author upon reasonable request.
